# Outcomes of children and young people with cerebral palsy receiving long‐term respiratory support: A systematic review

**DOI:** 10.1111/dmcn.70279

**Published:** 2026-04-23

**Authors:** Rajkumar Dhandayuthapani, Alasdair Campbell, Catherine M McDougall, Florian Gahleitner, Steve Cunningham, Don S Urquhart

**Affiliations:** ^1^ Department of Paediatric Respiratory and Sleep Medicine Royal Hospital for Children and Young People Edinburgh UK; ^2^ Department of Child Life and Health University of Edinburgh Edinburgh UK; ^3^ Centre for Inflammation Research University of Edinburgh Edinburgh UK; ^4^ Community Paediatrics Royal Hospital for Children and Young People Edinburgh UK

## Abstract

**Aim:**

To systematically review the current knowledge gap in, and impact of, long‐term respiratory support in children and young people (CYP) with cerebral palsy (CP).

**Method:**

We searched six databases from inception until 30th January 2025. Grey literature was also searched. Screening and data extraction using Covidence software, quality of evidence (Risk Of Bias In Non‐randomized Studies ‐ of Interventions), and certainty of evidence (Grading of Recommendation, Assessment, Development and Evaluation) grading were independently conducted by two reviewers, with a third reviewer resolving any lack of consensus.

**Results:**

Title and abstract screening (*n* = 1994) and full‐text reviews (*n* = 45) identified four studies (with moderate to severe risk of bias) for data extraction. Findings presented as a narrative synthesis from vote counting suggest improvement in sleep study parameters for CYP with CP on long‐term respiratory support and quality of life for their parents (low certainty of evidence).

**Interpretation:**

This review identified a significant knowledge gap in the use of long‐term respiratory support in CYP with CP and its impact on health‐related outcomes for such young people. This limits the information that can be offered when counselling parents/carers before initiating respiratory support in CYP with CP. Further research is needed to allow better service‐planning at national and local levels.

## Graphical abstract

Outcomes of children with cerebral palsy receiving long‐term respiratory support.

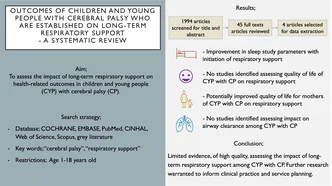

**This systematic review is commented by Cooper on pages** 1492–1493 **of this issue.**

**Abbreviations**
AHI: apnoea–hypopnoea index
CPAP: continuous positive airway pressure
CYP: children and young people
NIV: non‐invasive ventilation
QoL: quality of life
ROBINS‐I: Risk Of Bias In Non‐randomized Studies ‐ of Interventions
SDB: sleep‐disordered breathing

**What this paper adds**

- Respiratory support may improve sleep‐disordered breathing in children and young people (CYP) with cerebral palsy (CP).
- Evidence for respiratory support reducing respiratory infections in CYP with CP is limited.
- The effects of respiratory support on quality of life of CYP with CP are uncertain.

Cerebral palsy (CP) represents a heterogeneous condition characterized by an impairment of movement and/or posture, caused by a non‐progressive interference to the developing brain around the time of birth.^1^ With a steady incidence of 1 in 400 live births^2^ CP is the most common cause of disability among children and young people (CYP),^3^ with an estimated 30 000 CYP with CP in the UK.^2^ The Gross Motor Function Classification System (GMFCS) is used to classify the degree of functional mobility for CYP with CP. This system is categorized from levels I (mild impairment) to V (severe impairment), where GMFCS level V indicates severe restrictions in voluntary movements.^4^

The improved survival of CYP with CP to adulthood makes it the most common cause of life‐long disability in adults.^3^ However, CYP with CP have also been noted to have lower physical and health‐related quality of life (QoL) compared with typically developing peers.^5^ Several factors contribute to poor QoL among CYP with CP, including physical limitations due to pain and/or fatigue, sleep disturbance, and exclusion from social activities.^5^ Low socioeconomic status is a further potential contributing factor.^5^

Of note is the high risk of aspiration for CYP with CP, from gastro‐oesophageal reflex disease or unsafe swallow, with poor nutritional outcome contributing to poorer QoL for the child and their parents/carers. The Eating and Drinking Ability Classification System is a tool used to assess safety of swallow, scored on levels I to V, which indicate progressively unsafe swallow. Recurrent aspiration is a known contributor to an increased risk of respiratory illness, especially for CYP with CP classified in levels III to V of the Eating and Drinking Ability Classification System,^6^ and is commonly associated with sleep‐disordered breathing (SDB).^7^ Sleep disorders are common among CYP with CP, with a prevalence of 23% to 36%,^8^ particularly disorders in initiating and maintaining sleep (18%–27%).^9^ CYP with CP are also noted as being at a higher risk of SDB (13%–16%) than their typically developing peers (6%).^10^ SDB is an umbrella term encompassing obstructive sleep apnoea, central sleep apnoea, and hypoventilation. Multiple intrinsic and extrinsic factors contribute to the increased risk of SDB among CYP with CP, including tone and postural abnormalities, seizure disorders, and concomitant medications such as antiseizure and sedative medications.^11^

In addition to typical symptoms of snoring and pauses in breathing, CYP with CP may also show atypical features of externalizing symptoms such as irritability and agitation as well as internalizing symptoms (i.e. being withdrawn as a result of poor sleep quality).^9^, ^12^ CYP with CP often need frequent interventions overnight including repositioning to prevent pressure ulcers and/or to relieve symptoms of airway obstruction. Hence parents, who are often the primary caregivers for a CYP with CP, may have frequently disrupted sleep with a detrimental impact on their (parental) QoL as well.^13^ This, in turn, adds further stress to their caregiver responsibilities and has been named a contributing factor to depression in carers of CYP with CP.^14^, ^15^

CYP with CP can also be at significant risk of recurrent lower respiratory tract infections, which are also the leading cause of mortality^16^ for this patient group. Impaired airway clearance secondary to weak respiratory muscle function, poor posture, and coordination contributes to recurrent infections and atelectasis.^8^ Factors associated with recurrent hospitalization in CYP with CP include GMFCS level (level V has the highest risk), at least two courses of antibiotics or one respiratory hospital admission in the previous year, night‐time snoring, seizure, oropharyngeal dysphagia, and frequent respiratory symptoms (i.e. daily chesty cough or wheeze).^16^ Furthermore, CYP with CP are more prone to severe lower respiratory tract infection warranting critical‐care admission.^8^

A multidisciplinary approach is necessary to address the interrelated comorbidities that CYP with CP are prone to. Long‐term respiratory support, delivered as continuous positive airway pressure (CPAP) through a facemask or nasal mask, bilevel non‐invasive ventilation (NIV), or invasive tracheostomy ventilation, is a potential option used in the management of CYP with CP. Treatment of SDB, aiding airway clearance for those with recurrent lower respiratory tract infections or facilitating discharge from hospital to non‐acute care settings are all potential intended benefits in the use of respiratory support among CYP with neurodisability (such as CP).^17^, ^18^ Serial UK‐wide surveys undertaken over the past 30 years have demonstrated an exponential increase in the number of CYP overall (and CYP with CP) established on long‐term respiratory support.^19^, ^20^, ^21^, ^22^

The initiation of respiratory support for CYP with CP should involve discussions with the multidisciplinary team as well as detailed consultations with the young person with CP and their caregivers.^17^ Open discussions about the proposed indication and potential benefits of long‐term respiratory support, as well as potential difficulties and challenges, allow the parents to make an informed decision.^23^ Continued engagement with the family after establishing respiratory support, to review whether goals of treatment have been achieved, assesses potential benefits and mitigates any side effects. Such an approach allows for a shared consensus about the continued use of long‐term respiratory support.

The use of respiratory support among CYP with CP can be associated with an increased burden of care and financial implications for the carers and wider family.^24^ Furthermore, CYP with CP reportedly have poorer tolerance of long‐term respiratory support, resulting in a high failure rate.^25^ Despite the increasing use of long‐term respiratory support in CYP with CP, there is no published evidence, to date, systematically reviewing its impact. This makes it challenging to have an evidence‐based discussion about establishing respiratory support when counselling family members of CYP with CP. This review aimed to explore and characterize this knowledge gap, by systematically reviewing the impact of long‐term respiratory support (CPAP, NIV, invasive tracheostomy ventilation) on health‐related outcomes (sleep study parameters, hospital admission rates, antibiotic use, QoL, side effects, and mortality) in CYP with CP.

## METHOD

### Research question

The primary research question, developed using the Participant, Intervention, Comparator and Outcome (PICO) framework, was ‘What impact does home respiratory support have on health‐related outcomes of children and young people with CP, aged between 1 month and 18 years old?’

The primary outcomes considered were the impact of respiratory support on parameters of SDB using sleep studies (namely the apnoea–hypopnoea index [AHI], transcutaneous carbon dioxide levels, and oxygen saturation), frequency and duration of hospital admissions, and number of courses of antibiotics used for infections of the lower respiratory tract. The primary outcomes were prioritized to reflect real‐life practices and indications of long‐term ventilatory support for CYP with complex needs such as CP, for managing SDB and aiding airway clearance to reduce respiratory‐related hospital admissions.

The secondary outcomes considered were the impact of respiratory support on QoL for CYP with CP (owing to burden of symptoms and intervention), QoL of carers for CYP with CP (including ease and burden of caregiver responsibility, mental health, etc.), adverse outcomes (e.g. tolerance, complications, etc.), and mortality. Additional relevant outcomes encountered during the review were also considered, such as impact on functional status of the child with respiratory support.

### Protocol and registration

The reporting of this study was in accordance with the Preferred Reporting Items for Systematic reviews and Meta‐Analyses (PRISMA 2020).^26^ This study closely followed, with no deviation, the a priori protocol guided by the Preferred Reporting Items for Systematic review and Meta‐Analysis Protocols (PRISMA‐P)^27^ and Cochrane Handbook for Systematic Reviews of Interventions.^28^, ^29^, ^30^, ^31^ The study, along with the protocol, was registered on the PROSPERO database (CRD42025634543) as a systematic review of intervention.

### Eligibility criteria

Inclusion criteria for this review were (1) persons with a diagnosis of CP (as defined by the diagnostic criteria of each included study), (2) aged (inclusive) 1 month to 18 years old, (3) on respiratory support (provided as CPAP, bi‐level NIV, or invasive ventilation by tracheostomy) in a non‐acute care setting. For studies that included CYP with CP as part of a wider cohort of patients, only those with extractable data for CYP with CP were included. Exclusion criteria for this review included (1) cohorts of patients without a clearly defined diagnosis of CP, and/or (2) infants with CP who were on respiratory support for chronic lung disease of prematurity, also called bronchopulmonary dysplasia. No limitation was set on the design or language of the studies included. Where studies in a non‐English language were identified, attempts were made to translate them (using Google Translate) for analysis and data extraction.

### Search strategy

A comprehensive search was performed on the database (platforms) of Cochrane Central Register of Controlled Trials (CENTRAL), Excerpta Medica dataBASE (EMBASE) (Ovid), PubMed, Cumulative Index to Nursing and Allied Health Literature (CINAHL) (EBSCOhost), Web of Science (Clarivate's Web of Science), and Scopus (Elsevier). Grey literature identified through ProQuest was also included. Backward citation chasing of articles included for data extraction was also conducted to include title and abstract screening. The search strategy for each database was devised with assistance from the University of Edinburgh Library services (Appendix S1). The search strategy included database‐specific subject headings (and variants) relating to ‘cerebral palsy’ and ‘respiratory support’, with limitations on participants' ages between 1 month and 18 years. The age restriction was set to exclude articles discussing respiratory support in neonates for chronic lung disease. There were no restrictions on country or language of study. All databases were searched from inception up to and including 30th January 2025.

### Study selection and data extraction

The citations from each database were uploaded, as a research information systems file, to Covidence systematic review software (Veritas Health Innovation, Melbourne, Australia), accessed through the University of Edinburgh. Duplicates were removed automatically by the Covidence inbuilt de‐duplicator and manually by the reviewers. Two reviewers (RD and AC) independently reviewed the articles, first by title and abstract, and subsequently as full‐text articles. Lack of consensus, at the stage of title and abstract or full‐text review, was resolved independently by a third reviewer (DSU).

The review of selected full‐text papers identified eligible articles for data extraction. A data extraction template was drafted a priori, alongside the protocol, and adapted to fit the ‘data extraction 1’ tool of Covidence. Fields for data extraction included study methodology, baseline characteristics of participants, mode of respiratory support assessed, and outcomes being assessed. Data were extracted from eligible articles with the Covidence data extraction tool by RD and AC independently. Any differences were discussed between RD and AC to reach a consensus with input from the third reviewer (DSU) where this could not be reached.

### Risk of bias assessment

The risk of bias in the study methodology of articles eligible for data extraction was assessed using the Risk Of Bias In Non‐randomized Studies ‐ of Interventions (ROBINS‐I) tool.^32^ ROBINS‐I is the recommended tool of the Cochrane collaboration for assessing risk of bias in non‐randomized studies of interventions,^33^ which was the nature of all the studies included in this review. Seven domains were assessed to review bias relating to (1) confounding, (2) selection of participants, (3) classification of intervention, (4) deviation from intended intervention, (5) missing data, (6) measurement of outcome, and (7) selection of reported results. Studies were subsequently graded to be at ‘low risk’, ‘moderate risk’, ‘serious risk’, or ‘critical risk’ of bias. Studies lacking sufficient evidence for assessment of the domains were graded ‘No information’. ROBINS‐I assessment was conducted for all studies eligible for data extraction, independently by two reviewers (RD, AC) and discussed subsequently to resolve differences.

### Data synthesis

Given the substantial heterogeneity across the studies because of the type of respiratory support assessed and outcomes reported, a meta‐analysis was not suitable. A narrative synthesis of the data was, therefore, reported in line with the Synthesis Without Meta‐analysis guidance.^34^ Hence, no measure of heterogeneity or effect size was performed. Studies that reported similar predetermined outcomes were categorized together, with individual study findings described in summary tables. Vote counting, by majority where feasible, was used to assess direction of effect (i.e. positive association, negative association, or no association) of outcomes reported with respiratory support.^29^ The certainty of evidence for each outcome was assessed using the Grading of Recommendation, Assessment, Development and Evaluation (GRADE) framework^35^ by RD and AC independently with subsequent discussion to resolve any differences.

### Patient and public engagement

A patient and public involvement meeting, facilitated by the National Research Scotland patient and public involvement lead, was held with three parents and carers of children with CP in November 2024. The meeting identified the unique and individualized experiences of parents and carers of children (with CP) on long‐term respiratory support. Stakeholders also voiced their support and highlighted a need for further studies on the impact of such respiratory support on the child and the wider family, which we sought to address in this systematic review.

## RESULTS

### Study selection

The search of the databases identified 2540 citations, of which 1994 were screened for title and abstracts after de‐duplication (Figure S1). Forty‐five articles were eligible for full‐text reviews, of which four were selected for data extraction. The reasons for exclusion (*n* = 41) at the stage of full‐text review are listed in Figure S1. All studies selected for data extraction were single‐centre quantitative studies. Three of the studies included were retrospective cohort studies, with no comparator, while Mavi et al.^36^ was a cross‐sectional cohort study comparing CYP with CP planned for tracheostomy and CYP with CP who underwent tracheostomy (with or without invasive ventilation) (Table 1). Two studies reviewed the impact of tracheostomy ventilation, while the other two assessed a combination of CPAP and bi‐level NIV. The individual study design, country of study, and details of study cohort are described in Table 1. The studies collectively represented 54 CYP with CP on long‐term respiratory support. No studies with outcomes apart from the predetermined ones were identified through this review for inclusion.

**TABLE 1:** Studies eligible for data extraction, including study design, modality of respiratory support, and participant information.

Reference (country)	Study design	Study duration	Number (% of study cohort) of CYP with CP	Males (%)	Age, mean (SD) or median (range)	Mode of respiratory support
Borges et al.^37^ (Brazil)	Retrospective cohort study	N/A	10 (37)	Not stated	Not stated	Tracheostomy ventilation
Mavi et al.^36^ (Turkey)	Case–control study	N/A	15 (31)	Not stated	3.87 (3.25) months	Tracheostomy ventilation
Morrison et al.^38^ (Australia)	Retrospective cohort study	N/A	8 (57)	Not stated	8.68 (5.18) years	CPAP and bi‐level NIV
Grychtol and Chan^25^ (UK)	Retrospective cohort study	N/A	21 (100)	47.6	11.1 (1.7–16.1) years	CPAP and bi‐level NIV

*Note*: Ages reported in decimal years as in original studies. Abbreviations: CPAP, continuous positive airway pressure; CYP with CP, children and young people with cerebral palsy; N/A, not available; NIV, non‐invasive ventilation; SD, standard deviation.

### ROBINS‐I results

The risk of bias assessed using the ROBINS‐I tool is described in Figure 1,^25^, ^36^, ^37^, ^38^, ^39^ with all included studies having moderate to severe risk of bias. Methodological bias, particularly that due to baseline and time‐varying confounding, was noted in all the studies included in this review. There was insufficient information on confounding factors, or measures taken to control for confounding factors, in all the articles included. The criteria for participant selection and intervention were clearly defined in all studies, with minimal bias due to these domains. There were varying degrees of bias (or lack of information) relating to concordance with intervention, potentially missing data and/or measures to address missing values, and robustness in the measurement of relevant outcomes (Figure 1).

**FIGURE 1:**
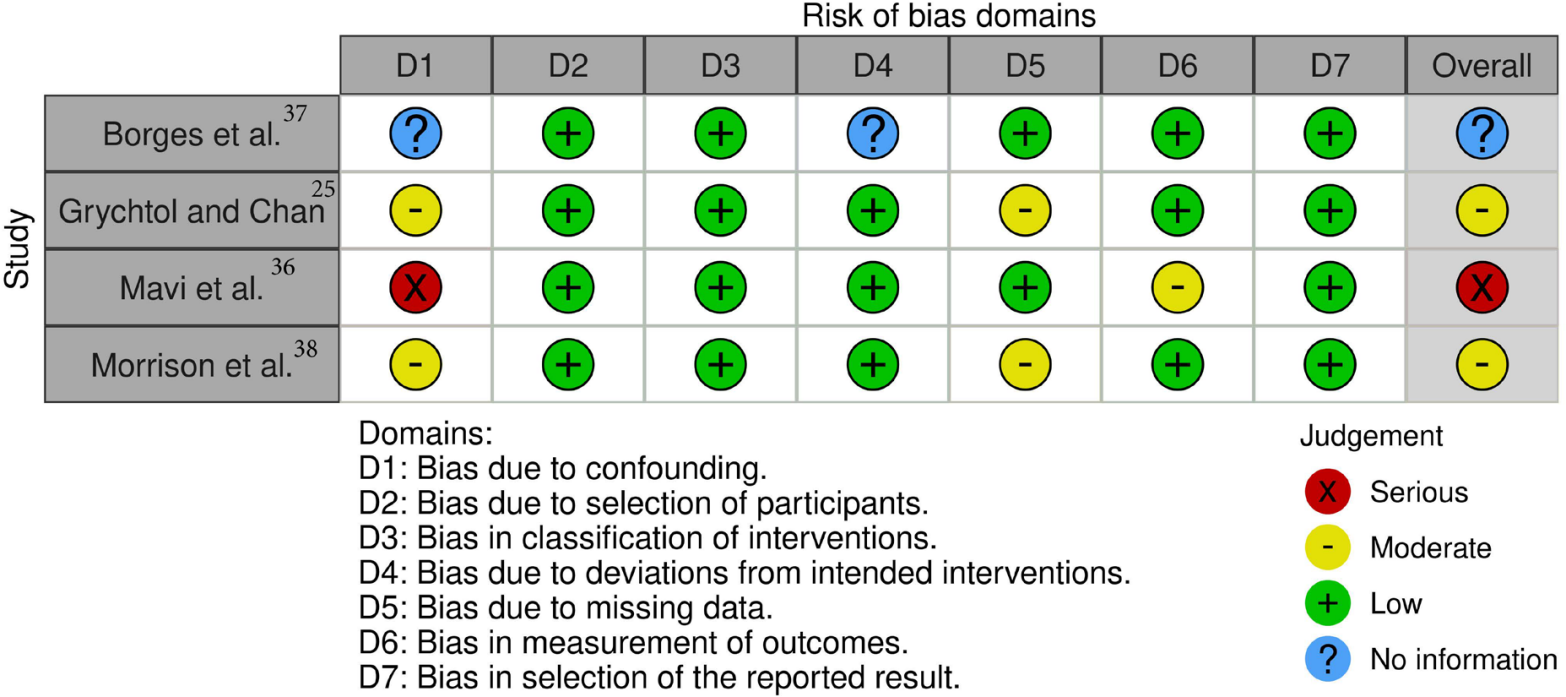
Risk of bias scoring using Risk Of Bias In Non‐randomized Studies ‐ of Interventions (ROBINS‐I) tool, for individual domains and overall study methodology.^25^, ^36^, ^37^, ^38^

### Sleep study parameters

Two studies^25^, ^38^ reported an improvement in SDB among CYP with CP after starting respiratory support (Table 2), although they had low certainty of evidence (Table 3). Data derived for CYP with CP from Morrison et al.^38^ note a reduction in AHI from a mean (range) of 75.1 (21.3–104.5), at baseline to 10.4 (3.9–15.2) after initiation of respiratory support. Grychtol and Chan^25^ reported a similar trend with reduction in obstructive AHI after starting respiratory support, for both CYP with CP who successfully established respiratory support and for those who failed to tolerate it after initiation, although the results were not amenable to extraction.

**TABLE 2:** Outcomes, individual study results, and direction of evidence.

Outcomes assessed	Study reference	Number of patients	Available data	Voting of direction of evidence	Risk of bias^a^
**Sleep study parameters**					
AHI	Morrison et al.^38^	3	Mean (range) change in AHI from 75.1 (21.3–104.5) events per hour at baseline, to 10.4 (3.9–15.2) events per hour after initiation of respiratory support.	Favours intervention	Moderate
	Grychtol and Chan^25^	16	Four of six CYP with CP who failed to establish long‐term respiratory support, had obstructive AHI <5 after initiation. The remaining two had residual obstructive events with obstructive AHI 5 and 18.6 events per hour. All 10 CYP with CP who successfully established long‐term respiratory support had AHI <5 events per hour.	Favours intervention	Moderate
ODI	Grychtol and Chan^25^	16	Three of six CYP with CP had ODI <5 events per hour, after initiating long‐term although they failed to establish in the long term, with other three having residual oxygen instability (ODI 12–21 events per hour). Eight of 10 CYP with CP, who successfully established long‐term respiratory support, had ODI <5 events per hour. The remaining two had residual oxygen instability owing to secretion‐related desaturation and type 2 respiratory failure not fully corrected with intervention.	Favours intervention	Moderate
**Quality of life of family members and carers of CYP with CP on long‐term respiratory support**					
Becks Depression Inventory scale^b^	Mavi et al.^36^	15	Median (range) score for CYP with CP on tracheostomy ventilation, 10 (0–29); similar to those with tracheostomy alone, 18 (13–28); *p* = 0.128.	No preference	Severe
Maslach Burnout Inventory^c^			Median (range) for emotional exhaustion favours tracheostomy ventilation. Cohort (5.5 [0–18]), compared with control (11.5 [0–19]); *p* = 0.002. Nil significant difference in median (range) score for depersonalization (1.5 [0–9] in CYP with CP on tracheostomy ventilation vs 3 [0–10] for control; *p* = 0.309) and personal achievement (26 [5–30] for CYP with CP on tracheostomy ventilation vs control 25 [13–32]; *p* = 0.917).	Favours intervention	Severe
Zarit Caregiver Burden Scale^d^			Median (range) score for mental tension (13 [7–28] for CYP with CP on tracheostomy ventilation vs control 19 [11–26]; *p* = 0.018) and irritability (5 [3–11] in CYP with CP on tracheostomy ventilation vs control 9 [4–11]; *p* = 0.021) favours intervention. Nil significant difference in scores for deterioration in social relation (3.5 [3–15] for CYP with CP on tracheostomy ventilation vs control 6 [3–16]; *p* = 0.113), economic burden (12.5 [4–18] in CYP with CP on tracheostomy ventilation vs control 13 [5–20]; *p* = 0.438) or dependence (7 [3–10] for intervention cohort vs control 8 [3–10]; *p* = 0.48).	Favours intervention	Severe

Abbreviations: AHI, apnoea–hypopnoea index; CYP with CP, children and young people with cerebral palsy; ODI, oxygen desaturation index.

^a^ Risk of bias assessed using Risk Of Bias In Non‐randomized Studies ‐ of Interventions (ROBINS‐I) tool.

^b^ Becks Depression Inventory scale; individual components (emotion, cognition, somatic and motivation) scored 0–3, with cumulative score (0–63) indicating normal (1–10), mild (11–16), borderline (17–20), moderate (31–40), or severe (>40) mood disturbance/depression.

^c^ Maslach Burnout Inventory; dimension of emotional exhaustion, depersonalization and personal achievement scored, with high score (for emotional exhaustion and depersonalization) or low score (for personal accomplishment) suggestive of burnout.

^d^ Zarit Caregiver Burden Scale; twenty‐two items across five domains (mental tension, irritability, deterioration in social relations, economic burden, and dependence) scored 1–5, with higher score suggestive of higher stress level.

**TABLE 3:** Certainty of evidence of outcomes assessed using Grading of Recommendation, Assessment, Development and Evaluation (GRADE).

Participants (studies)	Risk of bias	Inconsistency	Indirectness	Imprecision	Publication bias	Overall certainty of evidence
**Improving sleep‐disordered breathing (assessed with sleep study parameters)**						
19 (2 non‐randomized studies)	Serious^a^	Not serious	Not serious	Extremely serious^b^	Publication bias strongly suspected All plausible residual confounding would suggest spurious effect, while no effect was observed^c^	⨁◯◯◯ Very low^a^ ^,^ ^b^ ^,^ ^c^
**Quality of life of family members**						
15 (1 non‐randomized study)	Very serious^a^	Serious^b^ ^,^ ^c^	Not serious	Extremely serious^b^ ^,^ ^c^	Publication bias strongly suspected All plausible residual confounding would suggest spurious effect, while no effect was observed^c^	⨁◯◯◯ Very low^a^ ^,^ ^b^ ^,^ ^c^

^a^ Included studies are of moderate to serious risk of bias, owing to potential influence of confounding for the effect observed and the possibility of missing data.

^b^ Small number of participants in included studies, which is not powered to conclude observed effect with confidence.

^c^ Only a limited number of studies identified assessing the outcome of concern.

Grychtol and Chan^25^ also noted a general improvement in the oxygen desaturation index both for CYP with CP who successfully established and for those who could not tolerate respiratory support, with 11 out of 16 (68%) scoring an oxygen desaturation index value of fewer than five events per hour after initiating NIV. Grychtol and Chan^25^ also reported transcutaneous carbon dioxide levels within the normal range for all CYP with CP after starting respiratory support, except one who passed away before their first follow up.

### Hospital admission rates and antibiotic use

There were no studies identified that assessed the impact of respiratory support on hospital admission rates or the use of antibiotics in a clearly defined population of CYP with CP.

### Quality of life

One study^36^ identified potentially better QoL for mothers of children with CP who were tracheostomy ventilated (*n* = 15) compared with those who had tracheostomy alone (*n* = 11). The evidence was, however, of low certainty (Table 3). The study used validated self‐administered questionnaires of the Becks Depression Inventory, Maslach Burnout Inventory (subcategories of emotional exhaustion, depersonalization, personal achievement), and Zarit Caregiver Burden Scale (subcategories of mental tension, irritability, deterioration in social relationships, economic burden, and dependence).

Mothers of CYP with CP on tracheostomy ventilation were found to score lower for the emotional exhaustion (*p* = 0.002) component of the Maslach Burnout Inventory, and the mental tension (*p* = 0.018) and irritability (*p* = 0.021) components of the Zarit Caregiver Burnout Scale (Table 2). There were no significant differences in the other components of the Maslach and Zarit scales, or the Becks Depression Inventory.

There were no studies identified as part of this review, of a quantitative or qualitative nature, assessing the impact of long‐term respiratory support on the QoL of CYP with CP themselves.

### Adverse outcomes

#### Tolerance

Grychtol and Chan^25^ reported that 11 out of 20 (55%) CYP with CP failed to establish respiratory support, either because of poor tolerance during initial admission or subsequent poor adherence at home, with use of less than 2 hours per night and/or less than 50% of nights. Morrison et al.^38^ reported a failure rate of 2 out of 8 (25%) owing to poor tolerance.

Adherence to respiratory support, for CYP with CP who were successfully established, was similar between the studies of Grychtol and Chan^25^ and Morrison et al.^38^ Grychtol and Chan^25^ reported a median (range) use per night of 7.36 hours (3.22–13.45), and a percentage of the night used of 88% (55–100) for 9 out of 20 CYP with CP, while Morrison et al.^38^ reported a similar median (range) use per night of 8.72 hours (8.12–10.33) in 6 out of 8 CYP with CP.

Additionally, for CYP with CP who failed to establish respiratory support (11 out of 20), Grychtol and Chan^25^ described the median (range) of time used per night as 1.11 hours (0–2.55), and the percentage of night used as 51.5% (0–79).

#### Side effects

Common side effects noted from long‐term respiratory support included issues with the device and mask interface (10 out of 21), difficulty in managing secretions (3 out of 21), and abdominal symptoms such vomiting and bloating (2 out of 21).^25^

### Mortality

Mortality among CYP with CP on long‐term respiratory support was reported in two studies.^25^, ^37^ Grychtol and Chan^25^ reported two deaths out of 10 CYP with CP (20%) successfully established on non‐invasive respiratory support. One child died from a seizure during his sleep, unrelated to respiratory support use, while another died from multisystem failure secondary to gut malabsorption. Borges et al.^37^ reported an odds ratio of death among CYP with CP on invasive ventilation of 0.88 (95% confidence interval 0.19–4.24, *p* = 0.88), lower than the overall mortality rates for CYP who are invasively ventilated.

## DISCUSSION

The aim of this systematic review was to identify published evidence assessing the impact of long‐term respiratory support in CYP with CP, relating to effect on SDB, rates of hospital admissions, and antibiotic use. Additionally, the study sought to review the impact of long‐term respiratory support on QoL (of CYP with CP and their carers), as well as potential adverse outcomes and mortality. The review identified only four studies that were grouped as appropriate for available outcomes. Two additional studies^40^, ^41^ were considered for inclusion where CYP with CP formed a subset of the overall study population. However, on direct communication with the study authors, the data subset for CYP with CP was not available or amenable for extraction, which led to their eventual exclusion.

The evidence of the included studies (*n* = 4), however, was largely weak owing to the high risk of bias. Although it hampers the strength of conclusions drawn from this review, it maps the currently available evidence and highlights limitations relevant to clinical decision making. More importantly, it highlights significant gaps in the knowledge and evidence relating to the use of long‐term respiratory support, which has high cost and potentially high health impacts for an inherently high‐risk cohort of patients; these gaps could be addressed by future research.

SDB in CYP with CP can often be multifactorial, with residual symptoms often persisting despite surgical intervention: for example, adenotonsillectomy, such that CYP with CP may warrant long‐term respiratory support. This review identified two studies that showed an improvement in features of obstructive sleep apnoea measured as AHI or obstructive AHI on sleep studies. Although these findings are consistent, they are of low certainty of evidence given the high risk of bias of the included studies. The small number of participants in both studies precluded them from addressing confounding factors such as previous interventions of adenotonsillectomy, severity of impairment as classified by the GMFCS, other comorbidities such as seizures, and concurrent medications such as antiseizure medication or muscle relaxants. These confounders may potentially influence the extent of benefit seen with respiratory support in the resolution of SDB. Furthermore, CYP with CP may have multiple sleep disorders, which contribute to the night‐time sleep disturbances and subsequently affect daytime functioning. Hence, objective improvement in SDB measured with sleep study parameters may not always translate into subjective improvement in symptoms observed and does not guarantee tolerability either.

In addition to managing SDB, long‐term respiratory support is being increasingly used for aiding airway clearance and facilitating discharge from hospital.^17^ Although such benefits have been reported in children with other conditions such as spinal muscular atrophy^18^, ^42^, ^43^ or cystic fibrosis,^44^ there were no published studies investigating CYP with CP identified in this review. Zaman‐Haque et al.^45^ reported no benefit in hospital admission rates with NIV use among children with neurological conditions, although included diagnoses were not clearly defined. Morrison et al.^38^ reported a reduction in hospital admission rates for 5 out of 9 children with neurological conditions after initiation of long‐term respiratory support, although the overall hospital admission rates for the cohort remained similar before (*n* = 33) and after (*n* = 33) the initiation. However, the specific diagnoses of the children included were not given in this study either. The potentially conflicting evidence may be due to the small cohort of participants in the studies, resulting in them being insufficiently powered to identify meaningful benefits of the intervention. This further highlights the need for research to assess the benefits of long‐term respiratory support in reducing recurrent hospital admission of CYP with CP.

The use of long‐term respiratory support can have a significant impact on the QoL of CYP with CP, by improving their baseline QoL with resolution of SDB or by hampering their current QoL owing to disturbed sleep leading to poor tolerance. It forms an important aspect of discussion in consultations for the initiation of home ventilatory support. However, there were no studies identified that assessed the impact on QoL in CYP with CP on respiratory support. CYP with CP often have co‐existing learning disability, which potentially makes it challenging to evaluate such outcomes. Parent‐proxy measures are often used to assess QoL among CYP when direct assessment can be challenging, such as in CYP with CP. Hsiao et al.^46^ noted no difference in parent‐reported QoL for CYP with CP after treatment of obstructive sleep apnoea. However, the study reviewed both surgical interventions (adeno‐tonsillectomy) and CPAP, with difficulty in delineating the outcomes of CYP with CP who had respiratory support alone. Redouane et al.^41^ compared the impact of medical devices, such as long‐term ventilation and enterostomy tubes, on parent‐proxy QoL for CYP. However, further correspondence with the authors of the study identified only a small number of CYP with CP in the long‐term ventilation ‘arm’ to extract any meaningful and relevant data.

Several studies of CYP with other conditions on long‐term ventilation have reviewed QoL using self‐reported and/or parent‐proxy measures. Gonzalez et al.^47^ and Zhang et al.^48^ observed QoL of CYP (of all conditions) on long‐term ventilation to be lower than typically developing children. Gonzalez et al.^47^ and Mattson et al.^24^ also noted lower QoL among CYP (of all conditions) on home ventilatory support compared with CYP with other chronic conditions without ventilatory support, suggesting a negative association with home ventilation and QoL independent of the medical complexity of the CYP. A scoping review of qualitative studies by Mattson et al.^24^ described CYP on home ventilatory support reporting feelings of social exclusion resulting in lower QoL compared with typically developing CYP. However, the study also noted CYP not feeling restricted by ventilators and still having a good life despite them. These findings could potentially be extrapolated to CYP with CP in guiding discussions with families, although the unique challenges of CYP with CP caused by their inherent medical complexity must also be considered. Studies have also suggested parent proxy of QoL being scored consistently lower than self‐reported QoL by CYP,^24^, ^47^ which must be factored in, especially in CYP with CP where parent‐reported proxy will often be used in clinical settings.

The influence of the modality of respiratory support on QoL has been inconsistent: Zhang et al.^48^ reported lower QoL for CYP with invasive ventilation compared with NIV, whereas Gonzalez et al.^47^ reported no difference between the cohorts, except in self‐reported physical health where invasive ventilated CYP reported higher QoL than non‐invasive ventilated children. This might be due to the inherent heterogenicity of the study participants and their baseline function, where invasive ventilation might have allowed greater participation in activities from baseline thereby contributing to increased QoL.^24^, ^47^ Extrapolating these findings to CYP with CP also requires an acknowledgement of the patients' heterogeneity in the cohort, the focus on patient‐centred assessment, and the consideration of the potential impact that long‐term ventilation may be expected to have on an individual child/young person.

This review identified one study^36^ assessing the impact of long‐term respiratory support on parental QoL of CYP with CP, where invasive tracheostomy ventilation was found to reduce emotional exhaustion (Maslach Burnout Inventory) and irritability and mental tension (Zarit Caregiver Burden Scale) compared with carers of CYP with CP and tracheostomy alone. This, however, seems contrary to literature reviewing the QoL for parents of CYP with other conditions on long‐term respiratory support. Mattson et al.^24^ and Gonzalez et al.^47^ noted a greater financial burden for the family, as the parent (usually the mother) must often leave their professional job to fulfil the primary caregiver role. Furthermore, emotional components such as chronic fatigue and social isolation resulting from caregiver responsibility, frequently having to address medical emergencies, and potentially feeling guilty for neglecting the needs of other children may contribute to lower QoL for parents.^47^, ^49^ However, Gonzalez et al.^47^ reported parents feeling satisfied in their role as the caregiver for their child despite the added responsibility. Caring for their child might also validate their parental role and provide a sense of achievement, hence contributing to improved parental QoL.^36^ Mavi and colleagues^36^ also suggested the assurance of mechanical ventilation ensuring continued breathing for CYP with CP might explain the reduced maternal stressors and improved QoL. This highlights the complex interplay of patient heterogeneity, availability of social support, and different modalities of respiratory support, which influences the QoL of parents. Hence an individualized approach is necessary to assess QoL for CYP with CP and their family, at baseline and throughout their journey of establishing home respiratory support.^24^

The limited number of studies identified in this review noted a failure rate of 25% to 55% among CYP with CP in establishing long‐term respiratory support.^25^, ^38^ The failure rate in establishing CPAP by mask and/or NIV for CYP of all conditions ranges from 9% to 67%.^50^ Fauroux et al.,^50^ however, noted a varied definition for ‘failure to establish’ long‐term respiratory support. This is often defined as being due to poor tolerance, which is not well categorized in the paediatric population, thereby limiting generalizability. A suggested threshold of more than 4 hours' use per night for more than 50% of nights in a week for improved outcomes is advocated in adult patients, with an absence of a defined cut‐off in paediatric cohorts. Hence, a personalized approach to maximize use of respiratory support should be adopted rather than aiming for a fixed threshold.

A patient‐centred approach, as described in this review, is warranted to allow parents and caregivers (and the child where applicable) to share the making of an informed decision on the initiation of long‐term respiratory support. This often involves multiple discussions on the proposed goals of treatment, such as management of SDB or aiding airway clearance, the potential positive and negative impact it may have on the child as well as the parents/carers, and setting out clearly defined goals or outcomes to be reviewed after the set‐up of respiratory support.

This systematic review had several strengths and limitations. It involved a comprehensive search of six databases, with inclusion of articles from grey literature as well as studies found through citation‐chasing. There were no restrictions on country of study, although three studies of non‐English language were excluded during full‐text review, which might suggest language bias in the conduct of this review. The studies included for analysis were non‐randomized studies of interventions in nature, and were assessed using the Cochrane‐recommended ROBINS‐I tool. Given the heterogenous nature of respiratory support being assessed and of outcomes being reported, a meta‐analysis was not feasible and vote counting was used to assess direction of effect. Although recommended by the Cochrane collaboration as an alternative means of analysis and interpretation for systematic reviews,^29^ vote counting does not account for difference in sample size or the magnitude of effect reported.^29^ The reporting of certainty of evidence, using the GRADE framework, was also restricted given the small number of studies identified in this review, which potentially hampers the scoring of individual domains and skews overall certainty of evidence due to ‘lack of evidence’ as opposed to ‘evidence showing no effect’.

All studies included in this review were set in single centres in high‐income countries, which also limits the generalizability for CYP with CP in resource‐poor settings. The limited accessibility to specialist services such as long‐term ventilation and paediatric critical care for initiating long‐term respiratory support, and the higher burden of care on family members of CYP on long‐term respiratory support, with limited availability of community‐based support systems,^51^ limits the applicability of the findings of this review. Finally, the heterogeneity of respiratory support modalities being assessed, of the outcomes reported, and the lack of effect size prevented the formal reporting of publication bias.

## CONCLUSION

CYP with CP represent a challenging cohort of patients for successfully initiating long‐term respiratory support. The number of CYP with CP being set up on respiratory support, however, is increasing in the UK, with similar trends reported in other countries.^24^, ^48^ This systematic review identified limited published evidence assessing the potential impact of long‐term respiratory support on outcomes in CYP with CP, as well as the impact it has on their QoL and their wider family. A small number of studies, with high risk of bias, note the benefits of improvements in SDB measured using sleep study parameters, and improved QoL for parents of CYP with CP on long‐term respiratory support. However, the heterogeneity of these cohorts of patients and the modality of the long‐term respiratory support being assessed limit the certainty of evidence identified from this review. This potentially limits the evidence that we have available when discussing the initiation of long‐term respiratory support with CYP with CP and their caregivers. This systematic review identifies an evidence gap and highlights the need for research into the impact of respiratory support on the increasing number of CYP with CP who are being referred to sleep and long‐term ventilation services.

## FUNDING INFORMATION

The authors received no financial support for conducting or publishing this study.

## CONFLICT OF INTEREST STATEMENT

The authors have stated that they had no interests that might be perceived as posing a conflict or bias.

## Supporting information


**Figure S1:** PRISMA 2020 flow diagram detailing eligibility screening of articles.

**Appendix S1:** Search strategy for each database.

## Data Availability

The data that supports the findings of this study are available in the Supporting Information of this article.
