# Supplementary material for: Outcomes of children and young people with cerebral palsy receiving long‐term respiratory support: A systematic review
Source: Dev Med Child Neurol. 2026 Apr 23;68(11):1514–24. doi: 10.1111/dmcn.70279 (PMC13649304; doi:10.1111/dmcn.70279)
Supplement: Supplementary file 1 [file DMCN-68-1514-s001.pdf]

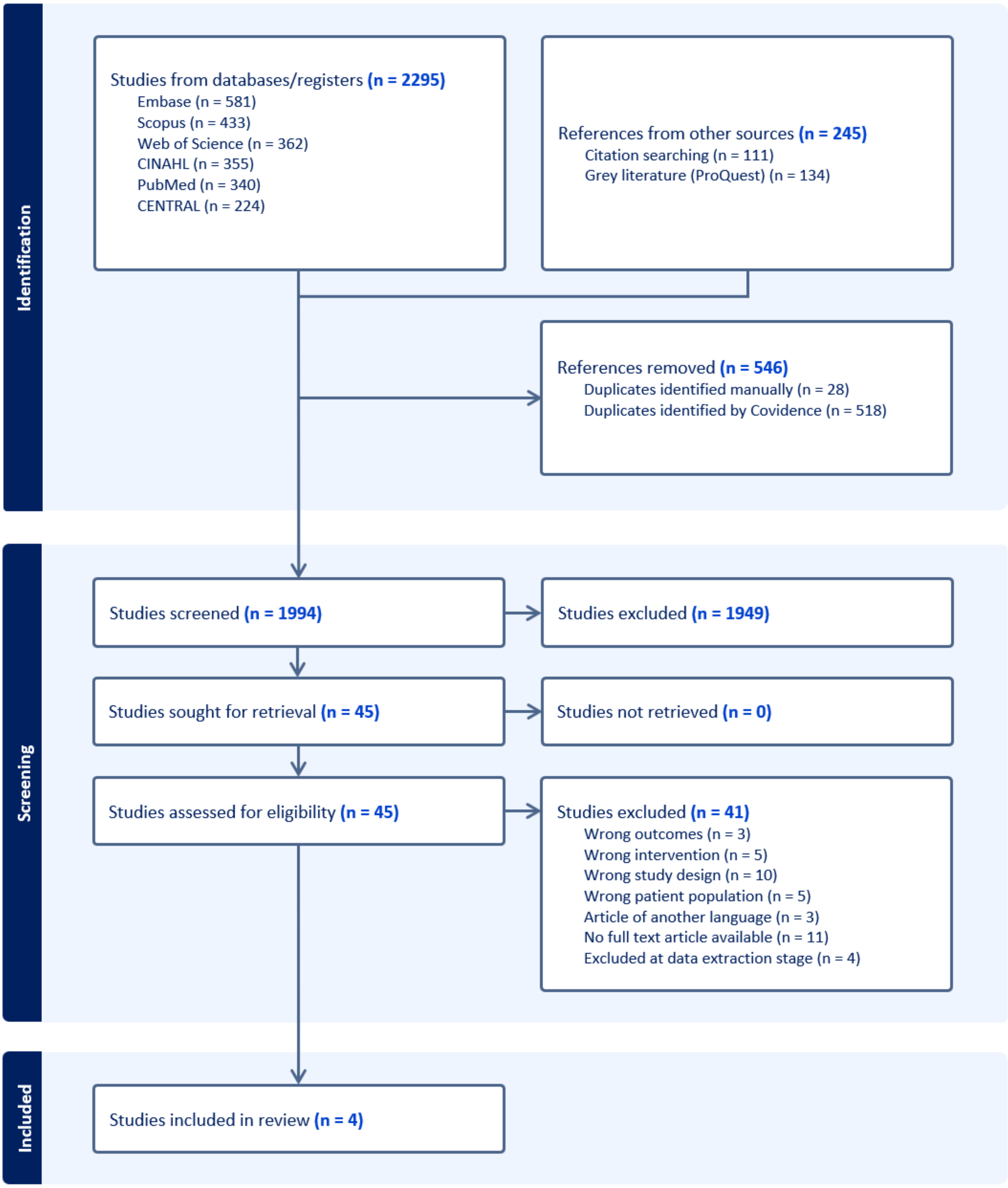

Figure S1: PRISMA 2020 flow diagram detailing eligibility screening of articles
