# Supplementary material for: Outcomes of children and young people with cerebral palsy receiving long‐term respiratory support: A systematic review
Source: Dev Med Child Neurol. 2026 Apr 23;68(11):1514–24. doi: 10.1111/dmcn.70279 (PMC13649304; doi:10.1111/dmcn.70279)
Supplement: Supplementary file 2 [file DMCN-68-1514-s002.docx]

**Appendix S1:** Search strategy for each database

| CINAHL; | | |
| --- | --- | --- |
| # | Query | Results |
| S20 | S19 NOT S18  Limiters - Age Groups: Infant, 1-23 months, Child, Preschool 2-5 years, Child, 6-12 years, Adolescence, 13-18 years, All Infant, All Child Expanders - Apply equivalent subjects Search modes - Find all my search terms | 354 |
| S19 | S9 AND S15 | 685 |
| S18 | S16 OR S17 | 4,852 |
| S17 | "chronic lung disease" | 2,270 |
| S16 | (MH "Bronchopulmonary Dysplasia") | 2,822 |
| S15 | S10 OR S11 OR S12 OR S13 OR S14 | 49,946 |
| S14 | Injury, infant brain OR Injury, f#etal brain | 4,168 |
| S13 | Disorder movement posture | 889 |
| S12 | Neurodevel* disor* OR Neurodisabi* OR Complex neur* disabi* OR Complex chronic disease | 18,358 |
| S11 | cerebral palsy OR CP | 27,711 |
| S10 | (MM "Cerebral Palsy") | 11,534 |
| S9 | S1 OR S2 OR S3 OR S4 OR S5 OR S6 OR S7 OR S8 | 68,231 |
| S8 | negative pressure ventil* | 893 |
| S7 | Tracheotomy ventil* OR trache*tomy ventil* OR tracheostomy ventil* | 2,554 |
| S6 | bilevel positive airway pressure OR BiPAP | 473 |
| S5 | continuous positive airway pressure OR CPAP | 8,219 |
| S4 | long term ventil* OR LTV | 3,315 |
| S3 | non-invasive ventil* OR niv OR nippv OR nppv OR non-invasive positive pressure ventilation | 3,393 |
| S2 | Respiratory supp* OR Home mechanical ventil* OR Ventil supp** OR Artificial ventil* | 45,538 |
| S1 | (MH "Respiration, Artificial+") | 40,005 |

| CENTRAL; | | |
| --- | --- | --- |
| ID | Search | Results |
| #1 | MeSH descriptor: [Respiration, Artificial] explode all trees | 9673 |
| #2 | ((respiratory near/3 supp*)) | 5110 |
| #3 | MeSH descriptor: [Noninvasive Ventilation] explode all trees | 585 |
| #4 | (Non-invasive ventilation) OR (Non-invasive ventilation) | 3795 |
| #5 | (Long term ventil*) OR (LTV) (Word variations have been searched) | 3459 |
| #6 | MeSH descriptor: [Continuous Positive Airway Pressure] explode all trees | 1943 |
| #7 | (continious positive airway pressure) OR (CPAP) (Word variations have been searched) | 6133 |
| #8 | (bilevel positive airway pressure) OR ("BiPAP") (Word variations have been searched) | 779 |
| #9 | MeSH descriptor: [Intermittent Positive-Pressure Ventilation] explode all trees | 353 |
| #10 | (tracheo* ventil*) (Word variations have been searched) | 1450 |
| #11 | MeSH descriptor: [Ventilators, Negative-Pressure] explode all trees | 41 |
| #12 | #1 OR #2 OR #3 OR #4 OR #5 OR #6 OR #7 OR #8 OR #9 OR #10 OR #11 | 23688 |
| #13 | MeSH descriptor: [Cerebral Palsy] explode all trees | 2392 |
| #14 | (cerebral pals*) | 6123 |
| #15 | MeSH descriptor: [Neurodevelopmental Disorders] explode all trees | 11538 |
| #16 | (neurodevel* disor*) OR (Neurodisabi*) OR (complex neural disabi*) | 2340 |
| #17 | (Disorder of movement NEXT posture) (Word variations have been searched) | 32 |
| #18 | #13 OR #14 OR #15 OR #16 OR #17 | 18718 |
| #19 | #12 AND #18 | 507 |
| #20 | MeSH descriptor: [Bronchopulmonary Dysplasia] explode all trees | 790 |
| #21 | ("chronic lung disease"):ti,ab,kw | 1653 |
| #22 | #20 OR #21 | 2336 |
| #23 | #19 NOT #22 | 356 |

| EMBASE; | | |
| --- | --- | --- |
| ID | Search | Results |
| 1 | exp artificial ventilation/ | 321939 |
| 2 | (Respir* adj4 supp*).af. | 26183 |
| 3 | (Home mechanical adj4 ventil*).af. | 1383 |
| 4 | (Ventil* adj4 supp*).af. | 28218 |
| 5 | (Artificial adj4 ventil*).af. | 208100 |
| 6 | 2 or 3 or 4 or 5 | 242621 |
| 7 | ((non-invasive adj4 ventil*) or NIV).af. | 23425 |
| 8 | ((long term adj4 ventil*) or LTV).af. | 4341 |
| 9 | exp continuous positive airway pressure/ | 13481 |
| 10 | exp noninvasive ventilation/ | 32357 |
| 11 | (Continuous positive airway pressure or CPAP).af. | 34198 |
| 12 | exp bilevel positive airway pressure/ | 2541 |
| 13 | (Bilevel Positive airway pressure or BiPAP).af. | 5292 |
| 14 | 7 or 8 or 9 or 10 or 11 or 12 or 13 | 80034 |
| 15 | (Tracheo* adj4 ventil*).af. | 10661 |
| 16 | (Negative-pressure adj4 ventil*).af. | 1226 |
| 17 | 1 or 6 or 14 or 15 or 16 | 364289 |
| 18 | exp cerebral palsy/ | 53218 |
| 19 | (cerebral palsy or CP).af. | 261464 |
| 20 | 18 or 19 | 261464 |
| 21 | exp neurodisability/ | 1115 |
| 22 | (Neurodevelop* adj4 disorder).af. | 13096 |
| 23 | neurodisability.af. | 2301 |
| 24 | 21 or 22 or 23 | 15374 |
| 25 | (disorder adj4 movement adj4 posture).af. | 87 |
| 26 | 20 or 24 or 25 | 276137 |
| 27 | 17 and 26 | 2696 |
| 28 | exp lung dysplasia/ | 17319 |
| 29 | exp chronic lung disease/ | 20568 |
| 30 | 28 or 29 | 36835 |
| 31 | (((artificial ventilation or ((Respir* adj4 supp*) or (Home mechanical adj4 ventil*) or (Ventil* adj4 supp*) or (Artificial adj4 ventil*)) or ((non-invasive adj4 ventil*) or NIV or ((long term adj4 ventil*) or LTV) or continuous positive airway pressure or noninvasive ventilation or (Continuous positive airway pressure or CPAP) or bilevel positive airway pressure or (Bilevel Positive airway pressure or BiPAP)) or (Tracheo* adj4 ventil*) or (Negative-pressure adj4 ventil*)) and (cerebral palsy or (cerebral palsy or CP) or (neurodisability or (Neurodevelop* adj4 disorder) or neurodisability) or (disorder adj4 movement adj4 posture))) not (lung dysplasia or chronic lung disease)).af. | 1945 |
| 32 | limit 31 to (infant <to one year> or child <unspecified age> or preschool child <1 to 6 years> or school child <7 to 12 years> or adolescent <13 to 17 years>) | 581 |

| **PubMed** | | |
| --- | --- | --- |
| Search number | Query | Results |
| 17 | #9 AND #15  Adolescent: 13-18 years, Child: 6-12 years, Infant: 1-23 months, Preschool Child: 2-5 years | 340 |
| 16 | #9 AND #15 | 681 |
| 15 | #10 OR #11 OR #12 OR #13 OR #14 | 64,932 |
| 14 | "Disorder movement posture" [tiab:~10] | 103 |
| 13 | Injury, foetal brain OR Injury, infant brain | 18,388 |
| 12 | "Neurodevelopmental disorder"OR complex neural disability | 9,922 |
| 11 | Cerebral palsy | 38,075 |
| 10 | "Cerebral Palsy"[Mesh] | 24,941 |
| 9 | #1 OR #2 OR #3 OR #4 OR #5 OR #6 OR #7 OR #8 | 135,491 |
| 8 | Negative pressure ventil* | 3,985 |
| 7 | Tracheostomy ventil* OR Tracheotomy ventil* | 8,053 |
| 6 | Bilevel Positive airway pressure OR BiPAP | 18,103 |
| 5 | Continuous positive airway pressure* OR CPAP | 20,048 |
| 4 | Long term ventil* OR LTV | 12,733 |
| 3 | Non-invasive ventilation OR Noninvasive ventilation OR NIV | 22,302 |
| 2 | "Respiratory support"[tiab:~4] OR "Home mechanical ventilation"[tiab:~4] OR "Ventilatory support" [tiab:~4] OR "Artificial ventilation"[tiab:~4] | 21,339 |
| 1 | "Respiration, Artificial"[Mesh] | 92,833 |

| Scopus | | |
| --- | --- | --- |
| No | Search string | Results |
| 1 | TITLE-ABS-KEY(artificial AND respiration) | 63 230 |
| 2 | TITLE-ABS-KEY ( respir* W/10 suppo* ) | 23 016 |
| 3 | TITLE-ABS-KEY ( home W/6 mechanical W/6 ventil* ) | 1 363 |
| 4 | TITLE-ABS-KEY ( ventil* W/6 suppor* ) | 22 090 |
| 5 | 1 OR 2 OR 3 OR 4 | 98 060 |
| 6 | ( TITLE-ABS-KEY ( non-invasive W/6 ventil* ) OR TITLE-ABS-KEY ( niv ) ) | 13 511 |
| 7 | ( TITLE-ABS-KEY ( long-term W/6 ventil* ) OR TITLE-ABS-KEY ( ltv ) ) | 6 384 |
| 8 | ( TITLE-ABS-KEY ( "continious positive pressure ventil*" ) OR TITLE-ABS-KEY ( cpap ) ) | 14 589 |
| 9 | ( TITLE-ABS-KEY ( "Bilevel Positive airway pressure ventil*" ) OR TITLE-ABS-KEY ( bipap ) ) | 1 435 |
| 10 | TITLE-ABS-KEY ( tracheo* W/6 ventil* ) | 5 133 |
| 11 | TITLE-ABS-KEY ( "Negative pressure ventil*" ) | 1 044 |
| 12 | 6 OR 7 OR 8 OR 9 OR 10 OR 11 | 38 789 |
| 13 | 5 OR 12 | 127 011 |
| 14 | TITLE-ABS-KEY ( "cerebral palsy" ) | 52 200 |
| 15 | ( TITLE-ABS-KEY ( "complex neur* disability" ) OR TITLE-ABS-KEY ( "Neurodevelop* disorder*" ) ) | 26 775 |
| 16 | ( TITLE-ABS-KEY ( injury W/6 fetal W/4 brain ) OR TITLE-ABS-KEY ( injury W/6 foetal W/4 brain ) OR TITLE-ABS-KEY ( injury W/6 infant W/4 brain ) ) | 1 738 |
| 17 | TITLE-ABS-KEY ( "disorder of movement and posture" ) | 80 |
| 18 | 14 OR 15 OR 16 OR 17 | 79 531 |
| 19 | 13 AND 18 | 433 |

| Web Of Science | | |
| --- | --- | --- |
| Number | Search terms | Results |
| 1 | TS=(respir* NEAR/5 suppor* OR Home mechanical ventilat* OR Ventilat* NEAR/5 suppor* OR Artificial NEAR/5 ventil*) | 32890 |
| 2 | TS=(non-invasive NEAR/6 ventil* OR NIV) | 12079 |
| 3 | TS=(long-term NEAR/6 ventil* OR LTV) | 5586 |
| 4 | TS=(“continious positive airway pressure" OR continuous positive pressure ventil* OR CPAP) | 15539 |
| 5 | TS=(Bilevel Positive airway pressure ventil* OR BiPAP) | 1390 |
| 6 | TS=(tracheo* NEAR/6 ventil* ) | 3885 |
| 7 | TS=(negative pressure ventil*) | 3789 |
| 8 | #7 OR #6 OR #5 OR #4 OR #3 OR #2 OR #1 | 66193 |
| 9 | TS=("cerebral palsy" ) | 39036 |
| 10 | TS=(Neurodevel* NEAR/6 disor* OR Neurodisab* OR complex neural disabil*) | 27949 |
| 11 | TS=(complex neuro* disabilit* OR neurodevel* disorder) | 42961 |
| 12 | TS=("disorder of movement and posture") | 20 |
| 13 | TS=(injury f$etal brain OR injury infant brain) | 11705 |
| 14 | #13 OR #12 OR #11 OR #10 OR #9 | 9133 |
| 15 | #14 AND #8 | 362 |

| ProQuest Dissertation and Theses | | |
| --- | --- | --- |
| Set# | Searched for | Results |
| S1 | "negative pressure ventil*" | 290 |
| S2 | ("continious positive airway pressure") OR ("continuous positive pressure ventil*") | 124 |
| S3 | tracheo* NEAR/4 ventil* | 1477 |
| S4 | "Bilevel Positive airway pressure" | 352 |
| S5 | (long-term NEAR/4 ventil*) | 1433 |
| S6 | (non-invasive NEAR/4 ventil*) | 1604 |
| S7 | (respir* NEAR/4 suppor*) OR ("Home mechanical ventilat*") OR (Ventilat* NEAR/4 suppor*) OR (Artificial NEAR/4 ventil*) | 19709 |
| S8 | [S1] OR [S2] OR [S3] OR [S4] OR [S5] OR [S6] OR [S7] | 22084 |
| S9 | noft("cerebral palsy") | 2404 |
| S10 | (Neurodevel* NEAR/4 disor*) OR Neurodisabi* OR "complex neural disabi* " | 526 |
| S11 | "disorder of movement and posture" | 114 |
| S12 | [S9] OR [S10] OR [S11] | 2932 |
| S13 | [S8] AND [S12] | 134 |
